# A phase II study of docetaxel in patients with metastatic squamous cell carcinoma of the head and neck

**DOI:** 10.1038/sj.bjc.6690715

**Published:** 1999-10

**Authors:** C Couteau, N Chouaki, S Leyvraz, D Oulid-Aissa, A Lebecq, C Domenge, V Groult, S Bordessoule, F Janot, M De Forni, J-P Armand

**Affiliations:** Institut Gustave Roussy, Department of Medecine, 39 rue Camille Desmoulins, Villejuif, 94805, France; Rhone Poulenc Rorer, Antony, France; Centre Pluridisciplinaire d'Oncologie, University Hospital, Lausanne, Switzerland

**Keywords:** docetaxel, head and neck carcinoma, phase II trial

## Abstract

This study was designed to evaluate the activity, safety and tolerance of docetaxel (D) in a selected population with metastatic squamous cell carcinoma of the head and neck (SCCHN). Twenty-four patients with no prior palliative therapy were enrolled and received D 100 mg m^−2^ by 1 h of infusion, every 3 weeks. All but two patients had been evaluated for efficacy on lung metastatic sites. No prophylactic administration of anti-emetics or growth factors was given. A pharmacokinetic study was performed in 22 patients. Twenty-one patients were assessable for response and 24 for toxicity. One hundred and four cycles were administered with a median of 4.5 (range 1–9) per patient. The median cumulative dose was 449 mg m^−2^. Partial responses were achieved in five patients with a median duration of 18.7 weeks (range 13.1–50.3). The overall response rate was 20.8% with a median duration of 11.0 weeks (range 2.4–52.6). The most frequent side-effect was neutropenia (79.2% grade IV) but with a short duration (median 4 days) and no febrile neutropenia. The incidence of moderate/severe fluid retention was 29.2% with one treatment discontinuation. Other toxicities (all grades) were common (skin 75%, asthenia 50%, infection 29.2%, nausea 16.7%, diarrhoea 12.5%, stomatitis 16.7%, vomiting 8.3% and HSR 8.3%). A mean clearance of 19.6 l h^−1^ m^−2^ and an area under the curve of 6.00 μg ml^−1^ h^−1^ was found in the pharmacokinetic analysis. Docetaxel is active in this selected population with metastatic SCCHN, with a good tolerance. © 1999 Cancer Research Campaign


*British Journal of Cancer* (1999) **81**(3), 457–462
© 1999 Cancer Research Campaign
Article no. bjoc.1999.0715

### A phase II study of docetaxel in patients with metastatic squamous cell carcinoma of the head and neck

**C Couteau[1], N Chouaki[1], S Leyvraz[3], D Oulid-Aissa[2], A Lebecq[2], C Domenge[1], V Groult[2], S Bordessoule[3], F Janot[1], M De Forni[1] and J-P Armand[1]**

[1]Institut Gustave Roussy, Department of Medecine, 39 rue Camille Desmoulins, 94805 Villejuif, France; [2]Rhone Poulenc Rorer, Antony, France; [3]Centre Pluridisciplinaire d'Oncologie, University Hospital, Lausanne, Switzerland

**Summary** This study was designed to evaluate the activity, safety and tolerance of docetaxel (D) in a selected population with metastatic squamous cell carcinoma of the head and neck (SCCHN). Twenty-four patients with no prior palliative therapy were enrolled and received D 100 mg $m^{-2}$ by 1 h of infusion, every 3 weeks. All but two patients had been evaluated for efficacy on lung metastatic sites. No prophylactic administration of anti-emetics or growth factors was given. A pharmacokinetic study was performed in 22 patients. Twenty-one patients were assessable for response and 24 for toxicity. One hundred and four cycles were administered with a median of 4.5 (range 1–9) per patient. The median cumulative dose was 449 mg $m^{-2}$. Partial responses were achieved in five patients with a median duration of 18.7 weeks (range 13.1–50.3). The overall response rate was 20.8% with a median duration of 11.0 weeks (range 2.4–52.6). The most frequent side-effect was neutropenia (79.2% grade IV) but with a short duration (median 4 days) and no febrile neutropenia. The incidence of moderate/severe fluid retention was 29.2% with one treatment discontinuation. Other toxicities (all grades) were common (skin 75%, asthenia 50%, infection 29.2%, nausea 16.7%, diarrhoea 12.5%, stomatitis 16.7%, vomiting 8.3% and HSR 8.3%). A mean clearance of 19.6 l $h^{-1}$ $m^{-2}$ and an area under the curve of 6.00 μg $ml^{-1}$ $h^{-1}$ was found in the pharmacokinetic analysis. Docetaxel is active in this selected population with metastatic SCCHN, with a good tolerance. © 1999 Cancer Research Campaign

**Keywords:** docetaxel; head and neck carcinoma; phase II trial

The estimated number of new cases of carcinoma of the head and neck (SCCHN) is over 60 000 per year in Western occidental countries (Landis et al, 1998). The location of the primary tumour varies, but the initial treatment remains surgery and/or radiotherapy if locoregional treatment is appropriate. Despite optimal treatment, 50% of patients develop locoregional recurrence, often in irradiated fields, and 30% have metastatic disease (Vokes et al, 1993; Armand et al, 1995).

Patients with recurrent or metastatic disease receive chemotherapy, but the objective response rate is about 10–50% for single agent treatments, with a short response duration ranging from 4 to 6 months. Proven effective drugs in SCCHN are cisplatinum, 5-fluorouracil (5-FU), methotrexate, bleomycin and more recently carboplatin and ifosfamide. The new drugs have not radically changed response rates. Therapeutic options for patients with incurable recurrent or metastatic disease are limited. Nevertheless, taxanes may improve results both as single agents and in association with other active drugs in SCCHN.

Docetaxel (Taxotere®) is a semisynthetic derivative of 10 deacetyl Baccatin III, an inactive non-cytotoxic precursor extracted from the European Yew (Bissery et al, 1991). Docetaxel is obtained after esterification of a side-chain. In vitro studies have demonstrated docetaxel activity on cell lines and murine or human tumour xenografts (Riou et al, 1992). Human SCCHN cell lines have proved to be highly sensitive to docetaxel even in platinum-refractory disease (Braakhuis et al, 1994). In vitro data indicate that there is no cross-resistance between docetaxel and 5-FU or cisplatinum in some cell lines.

Six phase I studies have determined neutropenia as the dose-limiting toxicity for docetaxel as 1-h infusion (Motti et al, 1992; Pazdur et al, 1992; Bissett et al, 1993; Burris et al, 1993; Extra et al, 1993; Tomiak et al, 1994). The recommended dose for phase II study was 100 mg $m^{-2}$ as a 1-h infusion, every 3 weeks.

In the phase I studies, objective responses were observed particularly in squamous cell carcinoma of the lung, head and neck and the other cancers of squamous origin. These arguments have justified phase II studies in SCCHN. We report one of these, in a selected population of patients with measurable metastatic disease.

*Received 20 April 1998*
*Revised 26 March 1999*
*Accepted 20 April 1999*

*Correspondence to:* J-P Armand

### PATIENTS AND METHODS

Patients were eligible for this study if they had histologically or cytologically proven SCCHN recurrent after initial treatment, locally advanced disease with no possibility of curative treatment by surgery and radiotherapy, or metastatic disease. Undifferentiated or non-keratinizing carcinoma and all tumours of the nasal and paranasal cavities or nasopharynx were excluded. Patients were allowed neoadjuvant chemotherapy prior to locoregional treatment but not within 12 months before entering the study.

Other criteria were: WHO performance status ≤ 1, adequate bone marrow, liver and renal functions. Patients with known or symptomatic brain or meningeal metastases, symptomatic peripheral

**Table 1** Pharmacokinetic protocols

| PK protocol number | Sampling times | | | |
|---|---|---|---|---|
| | Before infusion | During infusion | After infusion | After infusion (h) |
| I | Just before T0 | 5 min before end | 10 min after end | 2 h |
| II | Just before T0 | 30 min after start | 20 min | 3 h |
| III | Just before T0 | 5 min before end | 30 min | 4 h |
| IV | Just before T0 | 30 min after start | 1 hour | 5 h |

PK = pharmacokinetic.

neuropathy ≥ grade 2 according to the NCI criteria, or other serious illness or medical conditions were not eligible. Prior or concurrent treatment with colony-stimulating factors or continuous treatment with corticosteroids was not allowed. Signed informed consent was obtained from each patient before entering the study.

All patients had to have measurable lesions, with at least one diameter ≥ 2 cm, or multiple lung metastases with at least one lesion having two diameters ≥ 1 cm. At the Gustave Roussy Institute only patients with lung metastases were included in order to have clearly measurable disease. This excluded locoregional recurrences or locally advanced disease, which have imprecise limits making evaluation very subjective.

Each course of docetaxel was administered by intravenous (i.v.) infusion over 1 h, 100 mg $m^{-2}$ every 21 days. No anti-emetic or antibiotic prophylaxis was allowed. No systematic premedication was given to the patients. At the beginning of the study, steroids were not yet recommended to prevent hypersensitivity reactions and to decrease peripheral oedema. During the study a standard premedication was introduced: methylprednisolone 32 mg, orally 12 and 3 h before infusion, at the end of infusion, and 12, 24 and 36 hours after the end of infusion. Seven patients received corticosteroid premedication. Docetaxel was to be adjusted on the basis of toxicities, with dose reductions in patients who experienced grade 4 haematological toxicities (except grade 4 neutropenia), and for other grade 3 toxicities including skin, neurological and nausea/vomiting.

Computerized tomography (CT) scan was chosen as the method of evaluation as it is more precise and reproducible for the same lesion than X-rays. The response criteria was based on WHO definition. Response was defined as follows: complete response (CR), absence of all clinically and/or radiologically detectable disease; partial response (PR), greater than 50% reduction in the products of all measurable disease with no new lesions; stable disease (SD), less than 25% increase in tumour volume with no new lesions; and progressive disease (PD), greater than 25% increase in any tumour volume or the appearance of a new lesion. Evaluations were performed every 6 weeks.

Toxicity was evaluated according to National Cancer Institute (NCI) criteria. Patients were evaluated bi-weekly for laboratory parameters (blood cell counts, serum electrolytes and liver function tests), and every 3 weeks for clinical tolerance.

The duration of response and the time to progression were defined as the time elapsed from first administration to first documented progression or death. Survival was measured from the first day of treatment to death. All survival functions were computed using a Kaplan–Meier method (SAS software).

Pharmacokinetic (PK) blood sampling was performed at the time of first infusion. Blood samples were collected according to one of the four population pharmacokinetic protocols, summarized in Table 1.

Docetaxel concentrations were determined by high-performance liquid chromatography assay with UV detection at 225 nm. The method involved a solid-phase extraction step (C2 micro columns) using the Advanced Automated Sample Processor (AASP, varian). The extract was then automatically chromotographed on a C-18 reversed phase column. Pharmacokinetic parameters were estimated by Bayesian estimation using concentration time data for each subject and the previously defined population model as prior information. A three-compartment structural model with first-order elimination was used. Individual PK analysis was performed using the NON MEN programme. The analysis focused on docetaxel plasma clearance (CI) and the area under the curve (AUC) parameters.

### RESULTS

All 24 patients were entered into the study between December 1992 and July 1995. The characteristics of the population are summarized in Table 2. Of 24 patients enrolled and treated in the study, 21 were evaluable for response and 24 for toxicity. One patient was not eligible for response, because of insufficient tumour assessments. Two patients received only one cycle: one of these discontinued treatment because of renal failure after the first cycle, the other had skin toxicity related to the drug and was withdrawn after the first cycle. But all patients were included to calculate the intent to treat response rate. Nineteen patients (79.2%) had prior surgery and 20 (83.3%) previous radiotherapy at the primary tumour site. Only four patients had received neo-adjuvant chemotherapy of 5-FU–cisplatinum. All patients had good performance status (WHO criteria 0 or 1). The median age of the population was 60 years (range 46–69). The median disease-free interval between the initial diagnosis and first metastasis was 13 months (range 0–32 months). At the date of enrolment, 21 (87.5%) patients had metastatic disease: 17 had no local relapse and only metastatic disease, two had primary not excised with metastatic diffusion, two had locoregional recurrence and metastatic disease. The most common metastatic site was the lung: 22 patients (91.7%). All patients were carefully reviewed to differentiate between metastasis from SCCHN and a primary lung tumour, e.g. multiple sites of disease. No patients had isolated bone or liver metastasis. Two patients (8.3%) had only locoregional disease, with advanced hypopharyngeal primary (one with two synchronous head and neck tumours) with no prior treatment.

A total of 104 cycles were administered with a median of 4.5 per patient (range 1–9). Only one patient had a dose reduction after the first cycle (75 mg $m^{-2}$) due to grade IV neutropenia. In this

 *© 1999 Cancer Research Campaign*

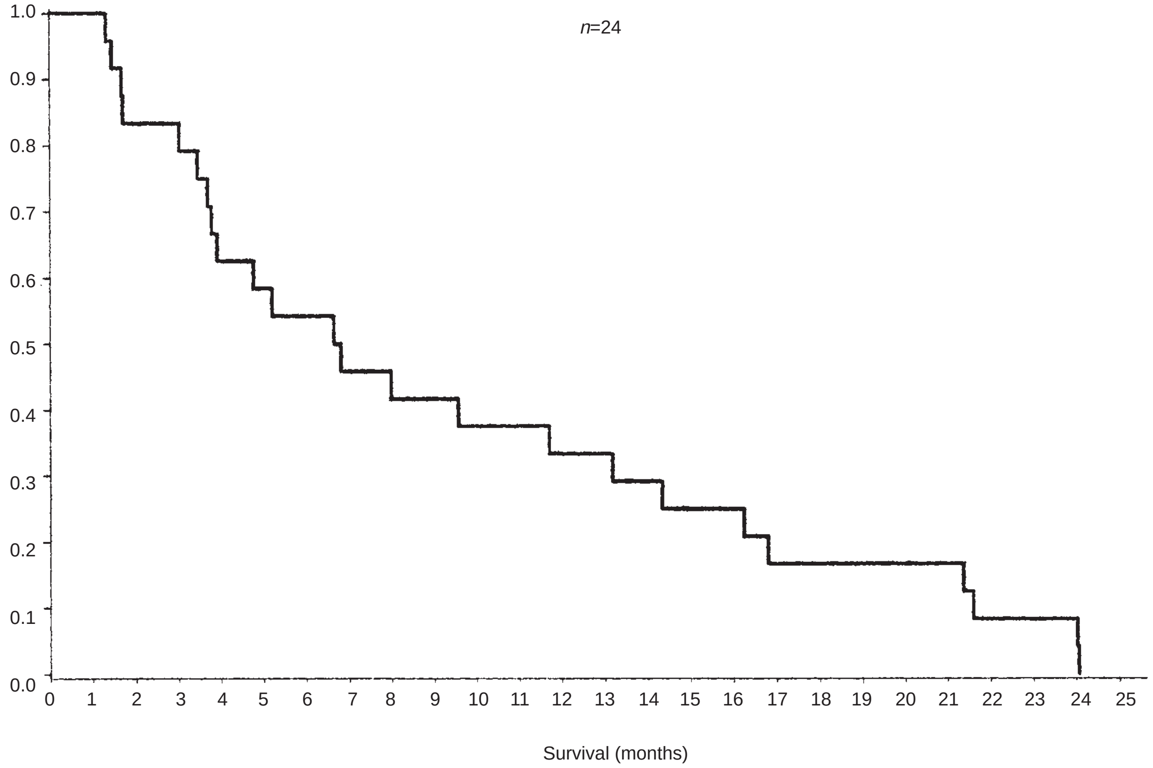

**Figure 1**

study 6/104 cycles were delayed more than 3 days but fewer than 7 days, and 2/104 cycles were delayed more than 7 days. The median cumulative dose was 449 mg $m^{-2}$ (range 99–916) with a relative dose intensity of 1.00 (range 0.78–1.04). Nine patients received at least six cycles. Treatment was discontinued because of toxicity in only two cases: one skin toxicity after the first cycle, and an association of skin toxicity and oedema after six cycles. One patient was lost to follow-up after four cycles. Only seven patients received preventive premedication for peripheral oedema since it was not systematically prescribed at the first infusion.

Individual pharmacokinetic analysis was performed in 22 patients. They had a mean age of 58.9 years (range 46–69) and a mean body surface area of 1.71 $m^2$ (range 1.32–2.00). Patients presented 1–4 quantifiable concentrations with actual sampling times ranging from 0.5 to 25 h.

The mean clearance in this population was 19.3 1 $h^{-1}$ $m^{-2}$ (range 7.7–30.8) and the mean area under the curve (AUC) was 6.00 μg $ml^{-1}$ $h^{-1}$ (range 3.28–12.50). The results obtained in this population with SCCHN were similar to those obtained in 577 patients with various type of tumours (Bruno et al, 1998). This PK study in a large population shows a mean clearance of 20.81 $h^{-1}$ $m^{-2}$.

Of the 24 patients enrolled and treated in the study, all were included in the efficacy analysis (intent to treat). Five patients had partial responses (20.8%), four with lung metastases and one with locally advanced disease. The overall response rate was 20.8% with 95% CI 7.1–42.2. For all patients the number of cycles given to obtain a response was two. The median duration of response was 18.7 weeks with a range (13.1–50.3) (cf Figure 1). The patient with the longest duration (50.3 weeks) had locally advanced disease. Median time to progression was 11.7 weeks (range 4.6–52.6). Median survival of the entire population was 6.7 months (range 1.3–24.0). Three of the responders had at least three metastatic sites involved. None of them had prior chemotherapy but the number was too small to conclude on this point. Their profiles and the duration of response respectively are the following: two patients had synchronous metastasis and primary tumour (one had a tonsillar and hypopharyngeal tumour with a cervical lymph node and contralateral supraclavicular

**Table 2** Population characteristics

| | |
|---|---|
| No of patients treated/eligible | 24/23 |
| Median age (range) | 60 (46–69) |
| Sex ratio: male/female | 23/1 |
| WHO performance status 0/1 | 14/10 |
| Prior surgery | 19/24 |
| Prior radiotherapy | 20/24 |
| Prior chemotherapy | 4/24 |
| Metastatic | |
| One site | 16/24 |
| Multiple sites | 5/24 |
| Locally advanced | 2/24 (8.3%) |
| Unknown | 1/24 (4.2%) |
| Sites of metastasis (*n* = 21) | |
| Lung | 91.7% |
| Bone | 12.5% |
| Lymph node | 12.5% |
| Pleura | 16.7% |
| Hypopharynx | 12.5% |
| Stage at diagnosis | |
| Stage I | 4 (16.7%) |
| Stage II | 2 (8.3%) |
| Stage III | 4 (16.7%) |
| Stage IV | 14 (58.3%) |

© 1999 Cancer Research Campaign

**Table 3** Toxicity in the 24 patients (worst grade per patient)

| | | |
|---|---|---|
| Haematotoxicity | | |
| Neutropenia | any grade | 95.8% |
| | grade IV | 79.2% |
| Thrombocytopenia any grade | | 4.2% |
| Anaemia grade III | | 12.5% |
| Febrile neutropenia | | 0% |
| Non-haematotoxicity any grade (grade 3–4) | | |
| Skin | | 75.0% (4.2%) |
| Alopecia | | 66.7% |
| Asthenia (severe) | | 50% (8.5%) |
| Fluid retention | | 45.8% |
| Incidence moderate/severe | | 29.2% |
| Infection | | 29.2% (4.2%) |
| Neurosensory | | 29.2% (4.2%) |
| Nausea | | 16.7% (0) |
| Stomatitis | | 16.7% (4.2%) |
| Diarrhoea | | 12.5% (4.2%) |
| Vomiting | | 8.3% (0) |
| HSR | | 8.3% (0) |
| Neuromotor | | 0 (0) |

HSR, hypersensitivity reaction.

disease (50.3 weeks of response duration); the second had synchronous mediastinal lymph nodes, lung metastasis, bone metastasis and primary tumour (16.0 weeks). The other three responders had: two lung metastases and a right adrenal mass (18.7 weeks); bilateral lung metastases (13.1 weeks); and lung and liver metastases with a left pleural effusion (40.5 weeks). For two patients progressive disease had been observed with the appearance of new lesions (skin metastasis for one and new lung metastasis for the second). The patient with locoregional recurrence progressed in a local site. The last two patients were withdrawn from the study despite partial response due to toxicity.

### Toxicity

Twenty-four patients were evaluable for toxicity (summarized in Table 3). Neutropenia was very frequent with an overall incidence of 95.8% of any grade and a majority of grade IV (79.2% of patients). However, median duration of neutropenia was short, 4 days (range 1–16), and growth factors were not required. Despite the high incidence of myelosuppression, there were no instances of febrile neutropenia, probably because of the short duration. In addition to haematological toxicity, one patient suffered from grade 3 infection but not neutropenia at the time. The most common non-haematological toxicity was skin toxicity (79.2%), including nail changes, onycholysis and cutaneous reactions. This toxicity was frequent but mild or moderate. Only one patient suffered from grade 3/4 (4.2%) skin toxicity. Two patients discontinued treatment due to skin toxicity (one after cycle 1 and the second after cycle six). Sixteen per cent of the patients suffered nausea and two patients from vomiting, all with grade 1 or 2. We observed hypersensitivity reactions in two patients. Two patients required premedication for subsequent cycles using dexamethasone and dexchlorpheniramine (H1 antagonist). They had no further hypersensitivity reactions. Fluid retention was reported in 45.8% of patients, and one patient discontinued the study due to fluid retention associated with skin toxicity. The incidence of moderate/severe fluid retention was 29.2%. Only seven patients were premedicated for oedema. The median cumulative dose to onset of fluid retention was 493 mg $m^{-2}$ and 596 mg $m^{-2}$ to moderate/severe. In the absence of premedication, only two patients had a weight gain of over 10% due to fluid retention.

One patient presented grade 3 neurotoxicity after six cycles of docetaxel. He had not received prior neurotoxic treatment, particularly cisplatinum. Only one death was related to a drug toxicity: pulmonary infection without neutropenia. Of 24 patients, all have subsequently died: 15 from progressive disease after the discontinuation of treatment, two from non-drug-related infection (for one patient, 1 month after the last cycle; for the second, 20 months after). Two patients died from cardiac disorders: one had a myocardial infarction at day 7 of cycle 5. He had a history of coronary bypass surgery and myocardial infarction and no relation to the study was established. For the second, it was unrelated because he died 85 days after the last cycle of congestive cardiac failure and he had a previous history of pulmonary embolism and high blood pressure. Four patients died from other non-drug-related causes.

The safety profile of docetaxel in this trial is the same as that observed in other phase II trials.

**Table 4** Patient characteristics in four phase II studies of docetaxel in SCCHN

| Dose of docetaxel (mg $m^{-2}$) | Catimel 100 | Dreyfuss 100 | Couteau 100 | Fujii 60 |
|---|---|---|---|---|
| No. of patients registered | 40 | 31 | 24 | 24 |
| No. of patients evaluable for response | 37 | 29 | 21 | 22 |
| Histology | | | | |
| Squamous cell carcinoma | 40 | 31 | 24 | 17 |
| Adenocarcinoma | – | – | – | 1 |
| Others | – | – | – | 6 |
| Disease extent | | | | |
| Local ± regional | 26 (66%) | 26 (84%) | 2 (8.7%) | Nd |
| Distant metastases | 11 (28%) | 3 (10%) | 17 (73.9%) | Nd |
| Locoregional + distant metastases | 3 (7%) | 2 (6%) | 4 (17.3%) | Nd |
| Prior therapy | | | | |
| Radiotherapy | 32 (82,5%) | 31 (100%) | 20 (83%) | Nd |
| Surgery | 24 (60%) | 26 (84%) | 19 (79%) | Nd |
| Induction chemotherapy | 10 (25%) | 10 (32%) | 4 (16.6%) | 16 (66.6%) |

Nd, not done in published paper.

 © 1999 Cancer Research Campaign

### DISCUSSION

The response rate in this study is much lower than the published results (Table 4) of three other studies. However, the selected population is very different, and this could explain the differences in results.

In the Japanese study (Fujii et al, 1995), docetaxel was administered every 21 or 28 days at a dose of 60 mg $m^{-2}$. Most importantly, this study included a mixed patient population: 17 had squamous cell carcinoma, two had salivary gland cystic adenocarcinoma, one had adenocarcinoma, three had undifferentiated tumours and one had mucinous squamous cell carcinoma. Among primary sites involved, five patients had nasopharyngeal tumour and four had a salivary gland primary. If we only take into account squamous cell carcinoma, the response rate, which was initially 5/22 (22.7%), becomes 2/17 (11%). Dose intensity was low (administration every 28 days), and the dose used was 60% of the recommended dose in Europe. However, the pharmacokinetics of docetaxel are known to be identical in European, American and Japanese populations (Tanigawara et al, 1996; Bruno et al, 1998).

The two other phase II trials in SCCHN (Catimel et al, 1994*a*; Dreyfuss et al, 1996) used the same schedule as ours: 100 mg $m^{-2}$ every 3 weeks. Once again the populations were different, and this could explain the higher response rates reported in these studies. The median response durations reported are consistent (about 6 months) and are comparable to those of other agents used separately or in combination.

Dreyfuss reported the results of a phase II study with 31 patients, of which 26 (84%) had advanced recurrent locoregional disease and three (10%) had metastatic disease, two had both (6%). As in our study there was a low incidence of peripheral oedema: five patients (13%). However, dose reduction due to neutropenia was frequent. The overall response rate was 42%. Of the 13 responders only one had metastatic disease: 1/13 (7%). Median response duration was 5 months (range 2–14).

The EORTC study included 43 patients (Catimel et al, 1994*a*). Forty were evaluable of whom 26 (66%) had locally advanced or recurrent disease, 11 (28%) metastatic, three both (7%). In this series, they did not find a difference in terms of response rate between the two populations: of patients with locally advanced disease, 8/25 (32%) had partial responses and 3/11 (27%) of patients with metastatic disease responded. However, numbers are rather low for comparison.

For other drugs, most published trials mixed locally advanced or recurrent disease. The first phase II of paclitaxel in SCCHN was conducted by the ECOG using a dose of 250 mg $m^{-2}$ administered over 24 h with granulocyte-stimulating factors between cycles (Forastiere et al, 1993). The overall response rate was 40%. Of the 27 patients enrolled, 17 had locoregional disease, six metastatic and four locoregional with distant diffusion. Ten objective responses were reported. Only two partial responses were seen in ten metastatic patients with lung spread. Response duration ranged from 1 to 5 months. An Ohio University study was proposed with the same schedule for 28 patients (Smith et al, 1995). No patient had metastatic disease at inclusion. Patients received three cycles of chemotherapy, followed by locoregional treatment. The authors reported an overall response rate of 23% with two complete and four partial responses, and a median response duration of 6 weeks to 3 months for the complete response. An Italian trial used a lower dose of paclitaxel (175 mg $m^{-2}$, over 3-h intravenous infusion) given only to patients with locoregional disease; the overall response rate was 20% without any complete response (Gebbia et al, 1996). In published trials, vinorelbine gives an overall response rate of 22% with no complete response (Gebbia et al, 1993*b*). Five partial responses were seen in a mixed population with no metastatic disease (two patients with no prior treatment and locally advanced disease, two with locoregional recurrence after radiotherapy and one with lymph node recurrence). Among the inhibitors of topoisomerase I, Topotecan has a response rate of 22% with a short duration (1–2.5 months) (Robert et al, 1994). Gemcitabine gave an overall response rate of 13% (7/61) in 61 patients (Catimel et al, 1994*b*). Three objective responses were observed in lung metastases. The median duration of response was 4 months (range 2–8.5). A phase II study of edertrexate (Schornagel et al, 1992) included 47 patients, of whom 15 (31%) had metastatic disease. The overall response rate was 5/47 (10.6%) with a median duration of 17 weeks (range 10–56).

Analysis of these results illustrates the difficulty of comparing studies even for one single drug. More recent trials have reported different response rates for the same drug, but this could be due to the different populations included. In our study, we decided to include only patients with lung metastasis because it is well known that SCCHN represents a constellation of tumours with various sensibilities to chemotherapy, such as laryngeal tumours, oral cavity primary and others. The evaluation of response rate in SSCHN can be more homogeneous if the same site of metastatic disease is chosen as was done in this study.

The other point of discussion for evaluation of a trial is the duration of response and the time spent on treatment of patients. In most published studies, including ours, the median duration of response is 5–6 months (20–24 weeks). Most patients received a median number of four cycles, then 12 weeks spent on treatment. For patients with symptomatic disease, time might be spent well on treatment if it produces a clinical benefit. For the others, particularly in metastatic disease, the benefit of treatment should be evaluated in terms of overall survival without treatment rather than in terms of percentage of response or median duration of response because the objective is not yet to cure. Evaluation of surrogate or new end points appear particularly important for the population with metastatic disease.

The role and the timing of treatment by chemotherapy is not well established. In our institute we have conducted an analysis of prognostic factors of response to cisplatinum-based chemotherapy in SCCHN (Recondo et al, 1991). Patients who had previous radiation therapy to the primary tumour had less response to cisplatinum infusion than those with only metastatic diffusion. The response rate was dependent on the extent of disease.

The expected response rate was 70% for neoadjuvant therapy, only 5% for a locoregional recurrence and 40% for metastatic disease. The following factors influenced cisplatinum chemotherapy response in univariate analysis: performance status, weight loss, length of disease-free interval, no bone metastasis, number of metastatic organs. In multivariate analysis, previous therapy and local relapse were the major variables which influenced the expected cisplatinum chemotherapy activity.

Taxanes could probably have a better response rate than alkylating agents in locoregional recurrence in previously irradiated fields. The difficulties: in reporting and comparing these studies are variations in tumour biology, adequate demonstration of recurrence and demonstrating that the duration of response is equivalent in relapsed and metastatic disease and is not dependent on the type of radiotherapy received. A good palliative treatment for local

© 1999 Cancer Research Campaign relapse for which radiochemotherapy combination remains a reasonable goal (Vokes et al, 1991; Gandia et al, 1993). Results of a phase II study for new drugs in head and neck cancer could identify compounds differently active in local and distant relapse.

This phase II study demonstrate that the toxicity of docetaxel in the population is acceptable with major neutropenia but no febrile neutropenia. Docetaxel has good activity in this population with metastatic disease.

### REFERENCES

Aapro M, Zulian G, Alberto P, Bruno R, Oulid-Aissa D and Le Bail N (1992) Phase I and pharmacokinetic study of RP56976 in a new ethanol free formulation. *Ann Oncol* **3** (abstract 208)

Armand JP and Couteau C (1995) Chemotherapy in head and neck treatment. *Eur J Cancer* **5**: 819–822

Bissery MC, Guenard D and Gueritte-Voegelein F (1991) Experimental antitumor activity of taxotere, a taxol analogue. *Cancer Res* **51**: 4845–4852

Bissett D, Setanoians A, Cassidy J, Graham MA, Chadwick GA, Wilson P, Auzannet V, Le Bail N, Kaye SB and Kerr DJ (1993) Phase I and pharmacokinetic study of taxotere administered as infusion. *Cancer Res* **53**: 523–527

Braakhuis BJM, Kagel A and Welters MJP (1994) The growth inhibiting effect of docetaxel (taxotere) in head and neck squamous cell carcinoma xenografts. *Cancer Lett* **81**: 151–154

Bruno R, Hille D, Riva A, Vivier N, Bokkel Huinnink W, van Oosterom A, Kaye S, Verweij J, Fossella F, Valero V, Rigas J, Seidman A, Chevallier B, Fumoleau P, Burris H, Ravdin P and Sheiner L (1998) Population pharmacokinetics/ pharmacodynamics of docetaxel in phase II studies in patients with cancer. *J Clin Oncol* **1**: 187–196

Burris H, Irvin R, Kuhn J, Kalter S, Smith L, Shaffer D, Fields S, Weiss G, Eckart J, Rodriguez G, Rinaldi D, Wall J, Cook G, Smith S, Vreeland F, Bayssas M, Le Bail N and Von Hoff D (1993) Phase I clinical trial of taxotere administered as either a 2 hour or 6 hour intravenous infusion. *J Clin Oncol* **11**: 950–958

Catimel G, Verweij J, Mattijssen V, Hanauska A, Piccart M, Vanders J, Franklin H, Le Bail N and Kaye SB (1994*a*) Docetaxel (Taxotere): an active drug for the treatment of patients with advanced squamous cell carcinoma of the head and neck. *Ann Oncol* **5**: 533–537

Catimel G, Vermorken JB, Clavel M, DeMulder P, Judsen I, Sessa C, Piccart M, Bruntsch U, Verweij J, Wanders J, Franklin H and Kaye SB (1994*b*) A phase II study of gemcitabine in patients with advanced squamous cell carcinoma of the head and neck. *Ann Oncol* **5**: 543–547

Dreyfuss AI, Clark JR, Norris CM, Rossi RM, Lucarini JW, Busse PM, Poulin MD, Thornhil L, Costello R and Posner MR (1996) Docetaxel an active new drug for squamous cell carcinoma of the head and neck. *J Clin Oncol* **14**: 1672–1678

Extra JM, Rousseau F, Bruno R, Clavel M, le Bail N and Marty M (1993) Phase I and pharmacokinetic study of taxotere given as a short intravenous infusion. *Cancer Res* **53**: 1037–1042

Forastiere AA, Neuberg D, Taylor IV SG, De Conti R and Adams G (1993) Phase II evaluation of Taxol in advanced head and neck cancer: an eastern cooperative oncology group trial. *J Natl Cancer Inst Monogr* **15**: 181–183

Fujii H, Sasaki Y, Ebihara S, Kida Y, Ichikawa G and Kaniyawa S (1955) An early phase II study of docetaxel (taxotere) in patients with head and neck cancer. *Proc Am Soc Clin Oncol* **14**: 298 (abstr 859)

Gandia D, Wibault P, Guillot T, Bensmaine A, Armand JP, Marandas P, Luboinski B and Cvitkovic E (1993) Simultaneous chemoradiotherapy as salvage treatment in locoregional recurrences of squamous head and neck cancer. *Head Neck* **15**: 8–15

Gebbia V, Testa A, Valenza R, Zerillo G, Restivo S, Ingria F, Cannata G and Gebbia N (1993) A pilot study of vinorelbine on a weekly schedule in recurrent and/or metastatic squamous cell carcinoma of the head and neck. *Eur J Cancer 29A* **9**: 1358–1359

Gebbia V, Testa A, Cannata G and Gebbia N (1996) Single agent paclitaxel in advanced squamous cell head and neck carcinoma. *Eur J Cancer* **5**: 901–902

Landis SH, Murray T, Bolden S and Wingo PH (1998) Cancer statistics, 1998. *Cancer J Clin* **48**: 6–29

Pazdur R, Newman RA and Newman BM (1992) Phase I trial of Taxotere: five days schedule. *J Natl Cancer Inst* **84**: 1781–1788

Ravdin P, Valero V, Nabholtz JM, Hortobagyi G, Oulid-Aissa D, Riva A, Houver C and Bellet R (1996) Efficacy of 5 day corticosteroid premedication in ameliorating taxotere induced fluid retention. *Proc Am Soc Clin Oncol* **14**: 115 (abstract 124)

Recondo G, Armand JP, Tellez-Bernal E, Domenge C, Belehradek M, De Vathaire F, Wibault P, Richard JM and Cvitkovic E (1991) Recurrent and/or metastatic head and neck squamous cell carcinoma: a clinical, univariate, and multivariate analysis of response and survival with cisplatin-based chemotherapy. *Laryngoscope* **101**: 494–501

Riou JF, Naudin A and Lavelle F (1992) Effects of taxotere on murine and human tumor cell lines. *Biochem Biophys Res Commun* **187**: 164–170

Robert F, Wheeler RH, Molthrop DC, Greene P and Chen S (1994) Phase II study of topotecan in advanced head and neck cancer, identification of an active new agent. *Proc Am Soc Clin Oncol* **13**: 281 (abstract 905)

Schornagel JH, Verweij J, De Mulder Ph, Cognetti F, Vermorken JP, Cappeleare, Armand JP, Wildiers J, Clavel M, Kirkpatrick A et al (1992) Phase II trial of 10 ethyl 10 deaza aminopterin, a novel antifolate, in patients with advanced and/or recurrent squamous cell carcinoma of the head and neck. *Ann Oncol* **3**: 223–226

Smith RE, Thornton DE and Allen J (1995) A phase II trial of paclitaxel in squamous cell carcinoma of the head and neck with correlative laboratory studies. *Semin Oncol* **22**: 41–46

Tanigawara Y, Sasaki Y, Otsu T, Fujii H, Kashiwura M, Sasaki T, Okuamura K and Taguchi T (1996) Population pharmacokinetics of docetaxel in Japanese patients. *Proc Am Soc Clin Oncol* **14**: 479 (abstract 1518)

Tomiak E, Piccart MJ, Kerger J, Lips S, Awada A, De Valeriola D, Ravoet C, Lossignol D, Sculier JP, Auzannet V, Le Bail N, Bayssas M and Kastersky J (1994) Phase I study of docetaxel administered as 1 hour intravenous infusion on a weekly basis. *J Clin Oncol* **12**: 1458–1467

Verweij J (1995) Docetaxel, an interesting new drug for the treatment of head and neck cancer and soft tissue sarcomas. *Anti Cancer Drugs* **6**: 19–24

Vokes EE, Awan AM and Weichselbaum RR (1991) Radiotherapy with concomitant chemotherapy for head and neck cancer. *Hematol/Oncol Clin N Am* **5**: 753–767

Vokes EE, Weichselbaum PP, Lippman SM and Hong WK (1993) Head and neck cancer. *N Engl J Med* **328**: 184–194

© 1999 Cancer Research Campaign
